# The Effect of University Students’ Levels of Knowledge about HPV Infection and the HPV Vaccine on Their Health Beliefs: Health Sciences Students

**DOI:** 10.3390/vaccines11061126

**Published:** 2023-06-20

**Authors:** Sibel Ergün

**Affiliations:** Faculty of Health Sciences, Department of Pediatric Nursing, Balıkesir University, Balıkesir 10145, Turkey; sibel.ergun@balikesir.edu.tr

**Keywords:** HPV vaccine, university students, knowledge levels, HPV infection, health beliefs

## Abstract

The aim of this study was to determine health sciences students’ levels of knowledge about HPV infection and the vaccine and their health beliefs, to compare them in terms of individual characteristics, and to examine the relationship between their knowledge of the HPV infection/vaccine and their health beliefs. The data of the study were collected from Health Sciences Faculty students through a face-to-face setting (n: 824). The data tools used in the study were the identification form, the health belief model scale for human papillomavirus infection and vaccination, and the human papillomavirus knowledge scale. The results showed that (1) although the students’ levels of knowledge about HPV infection and the vaccine were low, (2) they did perceive HPV infection to be a severe problem. According to the multilinear regression analysis performed, the main predictor of the perceived severity (β = 0.29; 95% Cl: 0.04, 0.07), obstacle (β = 0.21; 95% Cl: 0.01, 0.04), and sensitivity (β = 0.22; 95% Cl: 0.02, 0.06) subscales of the HBMS-HPVV was general HPV knowledge. It was also determined that as the students’ knowledge about HPV increased, their health belief levels regarding HPV infection and the vaccine increased as well (n: 824). In conclusion, for nurses and other healthcare professionals to be effective in informing individuals, they should have knowledge of HPV infection and the vaccine. In this context, the necessary education and advice about the importance of HPV infection and the vaccine should be provided to students receiving education in the field of healthcare.

## 1. Introduction

Human papillomavirus (HPV) is a prevalent viral infection of the genitourinary system [1]. HPV has many types, but most do not show any indication in the body; therefore, many people are not aware that they are infected with the virus. This lack of awareness has caused HPV to be considered a threat to public health [2]. HPV infection, which is seen in both females and males, can cause cancer [1]. HPV infections are divided into two groups in terms of the genital carcinogenic properties. The first group includes low-risk HPV types. These are HPV 6 and 11 types, which cause cervical lesions and genital warts. The second group includes high-risk HPV types. High-risk HPV 16–18 are the types that cause squamous carcinoma of the anus, vagina, vulva, penis, and cervix. High-risk HPV types were detected in 99% of cervical precancerous tissues. It has been reported that type 16 causes approximately 50% of cervical cancers globally, and that types 16 and 18 constitute 66% of cervical cancers. In addition, five high-risk types (31, 33, 45, 52, and 58) were found to be responsible for 15% of cervical cancers and 11% of all HPV-related cancers [2]. According to the Catalan Institute of Oncology (ICO) and the International Agency for Research on Cancer (IARC) HPV Information Center’s HPV-related diseases 2019 report for Turkey, the prevalence of HPV types 16 and 18 in all women over the age of 15 in Turkey varies between 4.2% and 67.6% [3]. The World Health Organization (WHO) has reported that levels of HPV infection are spreading rapidly, and that the morbidity and mortality rates of cervical cancer will increase [1]. The World Health Organization (WHO) states that cervical cancer is the fourth most common cancer among women worldwide. In 2018, more than 300,000 women died from cervical cancer, with nearly 90% of these deaths occurring in low- and middle-income countries [4]. According to Turkey’s health statistics for 2020, the incidence of cervical cancer in Turkey is 4.3/100,000, and among the types of gynecological cancer type, it is in the third place after endometrial and ovarian cancers [5]. Other than cervical cancer, HPV is also responsible for 91% of anal cancers, 75% of vaginal cancers, 69% of vulvar cancers, and 63% of penile cancers [2]. In this context, protection from HPV infection should be given priority in healthcare services worldwide, as should correct and early diagnosis [1,6]. In 2020, the World Health Assembly adopted a global strategy to eradicate cervical cancer. This strategy aims to administer three doses of the HPV vaccine to 90% of the population globally by 2030. In doing this, it has been stated that all countries need to improve their health programs on this subject [7]. However, it has been reported that there is insufficient public awareness about HPV infection, transmission routes, vaccination, and screening programs worldwide [8]. Vaccination and screening programs are recommended for protection against HPV infection [9]. The HPV vaccine itself is recommended for use in males and females between the ages of 11 and 26 [10]. University students represent a growing population within this age group [11]. Secondary school and university students are also the populations most at risk from HPV infection [12]. Receiving the vaccine during adolescence without having been exposed to HPV infection provides the best protection [13].

The HPV vaccine is not included in the national vaccination schedule in Turkey [9]. In order for nurses and other healthcare professionals to be effective in informing individuals about HPV, they need to have knowledge of HPV infection and the vaccine. In this context, the necessary education and advice about the importance of HPV infection and the vaccine should be provided to students receiving education in the field of healthcare [14]. In a study conducted with health sciences students in Turkey, the level of knowledge of the students about cervical cancer was examined and it was determined that 55.2% of the students had sufficient knowledge, 35.4% had a partially sufficient knowledge level, and 9.4% had insufficient knowledge about cervical cancer. This insufficient awareness about HPV infection and the vaccine in Turkey is considered to be due to the high cost of the vaccine and the lack of free HPV vaccinations in the national immunization program. Therefore, policies for adding the HPV vaccine to the national vaccination program should be implemented through a well-designed and sustainable awareness program. If basic information about HPV infection and the vaccine is provided to healthcare students in their undergraduate education period, their awareness will be raised, and they will contribute to raising social awareness through the information they provide to the public during their professional lives [15].

In light of all this information, the present study aimed to examine health sciences students’ knowledge of HPV infection and the vaccine and their health beliefs. Studying this group is important because healthcare students play a vital role in educating the public about HPV infection/vaccination during their training and after graduation. In addition, healthcare students are also parents of the future. In this context, their awareness of HPV infection and the vaccine may play a significant role in their making positive decisions about vaccinating their own children in the future [16].

**Figure 1:**
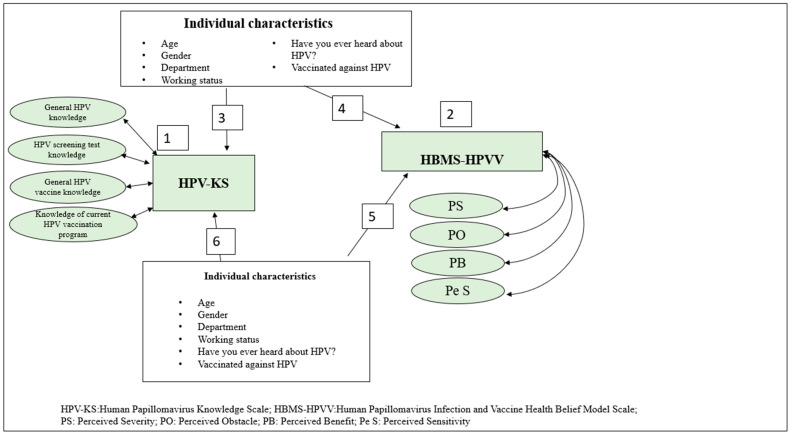
Research model.

The study aimed (1) to determine the students’ levels of knowledge about HPV infection and the vaccine and their health belief levels, (2) to compare their individual characteristics with their knowledge of HPV infection and the vaccine and their health belief levels, and (3) to identify the relationship between their individual characteristics and their HPV infection/vaccine knowledge and their health beliefs. In line with these purposes, answers were sought to the following research questions:

1. What are the students’ levels of knowledge about HPV infection and the vaccine?
2. What are the students’ health beliefs regarding HPV infection and the vaccine?
3. What individual characteristics of the students create a significant difference in their levels of knowledge of HPV infection and the vaccine?
4. What individual characteristics of the students create a significant difference in their health beliefs regarding HPV infection and the vaccine?
5. Does at least one of the students’ characteristics predict their health belief regarding HPV infection and the vaccine?
6. Does at least one of the students’ characteristics predict their level of knowledge about HPV infection and the vaccine? (Figure 1).

## 2. Methods

### 2.1. Study Design and Sample

The study had a descriptive and cross-sectional design. The data were collected through face-to-face interviews between 4 April and 1 June 2022.

The population of the study consisted of students studying at a Health Sciences Faculty of a state university located in the west of Turkey. The study population consisted of 914 faculty members who studied in the departments of Nursing (514 students), Midwifery (273 students), and Physiotherapy and Rehabilitation (PTR) (127 students). A sampling calculation was not made, and the aim was to access the whole population. However, 54 students did not volunteer to participate in the study. Since 24 students did not attend the classes during the study period and could not be reached, their forms could not be filled in. In addition, 12 students were not included in the evaluation because their forms were not complete. The study was completed with the participation of 824 students. Thus, 90.1% of the population were accessed.

The data were collected through a face-to-face setting. The purpose and significance of the study were explained before the class hour, and the questionnaire forms were distributed and collected by the researcher. The data were collected in the classrooms of the students under the supervision of the researcher. Students were first informed about the content and objectives of the study. Questionnaires were distributed to students who voluntarily agreed to participate in the study. Students answered the questionnaires in 10–15 min. After answering all the questions, the students submitted their questionnaires. The researcher was with the students during this process and ensured that they each answered individually.

### 2.2. Measures

Identification Form: This form, which was developed by the researcher in line with the literature, consisted of questions about the students’ age, gender, year of study, department, marital status, having heard of HPV prior to the study, and being vaccinated against HPV [15,16]. 

Human papillomavirus infection and vaccine health belief model scale (HBMS-HPVV): This scale, consists of 14 items under four subscales: perceived severity (PS) (four items); perceived obstacle (PO) (five items); perceived benefit (PB) (three items); and perceived sensitivity (Pe S) (three items). The scale items are scored on a four-point Likert-type scale ranging from “none” (1 point) to “very much” (4 points). It was determined that the internal consistency coefficients of the subscales varied between 0.71 and 0.78 [17]. In the present study, this value ranged from 0.76 to 0.92. 

Human papillomavirus knowledge scale (HPV-KS): This scale, investigates whether the respondent has heard of HPV infection and the vaccine, and the HPV screening test and their level of knowledge. The scale items are responded to with “Yes”, “No”, and “Don’t know”. In the evaluation phase, each correct answer is given 1 point, while incorrect and “Don’t know” answers are scored as 0 points. The score obtained from the HPV-KS ranges from 0 to 33. It was found the internal consistency coefficients of the subscales to be between 0.72 and 0.96 [18]. In the present study, these coefficients were found to vary between 0.61 and 0.85.

### 2.3. Statistical Analysis

The study data were analyzed using the IBM SPSS Statistics 21 package software. In the analysis of basic descriptive statistics, number, percentage, and mean were used. In the normality analyses of the data, skewness and kurtosis values were evaluated. As the data showed a normal distribution and were homogeneous, statistical analysis was performed with parametric test methods. The results were evaluated with a 95% confidence interval and at a significance level of *p* < 0.05. Then, the sociodemographic variables of age, gender, department, employment status, being aware of HPV before this study, and being vaccinated against HPV were accepted as independent variables, and multilinear regression analysis was performed with the subscales of the HBMS-HPVV. Likewise, the sociodemographic variables of age, gender, department, employment status, having heard of HPV, and being vaccinated against HPV were accepted as independent variables, and multilinear regression analysis was performed with the subscales of HPV-KS. 

### 2.4. Ethics

Approval was received from the Ethics Committee (date: 8 March 2022, No: 2022/32). Permission for the study was also obtained from the Dean of Health Sciences (4 April 2022-E.130507).

## 3. Results

### 3.1. Sociodemographic Characteristics

The mean age of the students participating was 20.92 ± 1.96. It was determined that 82.2% of the students were female, 54.6% were nursing department students, and 90.7% were not employed in any job, 100.0% were single. It was found that 71.6% of the students had heard of HPV before, and 93.1% had not received the HPV vaccine (Table 1).

### 3.2. Comparison of Health Beliefs about HPV Infection/Vaccination and HPV Knowledge Levels according to Sociodemographic Characteristics

The sociodemographic variables of the study sample and their mean scores obtained from the subscales of the HBMS-HPVV are compared against the mean scores obtained from the subscales of the HPV-KS in Table 1. The mean subscale scores of the female participants from the HBMS-HPVV (PS, PB, Pe S) were found to be higher compared to the mean scores of the male participants, and a statistically significant difference was found only with the PB subscale (*p* < 0.001). In addition, the female students’ mean scores obtained from all subscales of the HPV-KS were found to be higher than those of the male students, with a statistically significant difference (*p* < 0.001).

All the HBMS-HPVV subscale mean scores and the HPV-KS subscale scores of the PTR students were determined to be significantly lower than the scores of the midwifery and nursing students (*p* < 0.001).

The current HPV vaccination program knowledge subscale mean score of the students who were not employed was found to be higher than that of the students who were employed, and the difference was statistically significant (*p* < 0.001).

All of the HBMS-HPVV subscale mean scores and HPV-KS subscale scores of the students who had heard of HPV prior to the study were determined to be higher than the scores of those who had not heard of it, and a statistically significant difference was found (*p* < 0.001).

The HBMS-HPVV subscale (PS, PO, PB, Pe S) mean scores of the students who were vaccinated against HPV were determined to be higher compared to those who were not vaccinated, and a statistically significant difference was found only with the subscales of PS and Pe S (*p* < 0.001). It was also found that the students who were vaccinated scored higher on the subscale of PO, but there was no statistically significant difference (*p* > 0.05). In addition, all of the HPV-KS subscale mean scores of the students who were vaccinated were determined to be higher, and a statistically significant difference was found only with the subscale of general HPV knowledge (*p* < 0.001) (Table 1).

### 3.3. Health Beliefs of Students Regarding HPV Infection/Vaccination and HPV Knowledge Levels

Table 2 shows the mean values obtained from the scales analyzed. Regarding the subscale mean scores of the HPV-KS, the mean score for general HPV knowledge was determined as 7.88 ± 4.08, for HPV screening test knowledge as 1.55 ± 1.51, for general HPV vaccine knowledge as 2.10 ± 1.67, for knowledge of the current HPV vaccination program as 1.31 ± 1.39, and the total scale mean score was found to be 12.16 ± 7.21. As regards the subscales of the HBMS-HPVV, the mean score for Pe S was found to be 2.50 ± 0.85, for PB was 2.67 ± 0.89, for Pe S was 2.35 ± 0.82, for PO was 1.88 ± 0.62, and the total scale mean score was determined as 32.16 ± 8.91.

### 3.4. Multiple Linear Regression Analysis of Factors Influencing Students’ Health Beliefs about HPV Infection/Vaccination

In the study, multiple regression analysis was used to determine the predictive capacity of independent variables (age, gender, department, working status, having heard of HPV, HPV vaccination status) according to the four dimensions of the HBMS-HPVV. The results of the analysis are shown in Table 3. The variables explained 26% of the PS subscale score (F = 42,867; *p* < 0.000), and the best positive predictor was “Have you ever heard about HPV?”. For every one-unit increase in “Have you ever heard about HPV?”, the score for the PS subscale increased by 0.42. The variables explained 0.6% of the PO subscale score (F = 8.783; *p* < 0.000). Among all of these variables, and using the β coefficient, “Have you ever heard about HPV?” was the best positive predictor. For every one-unit increase in “Have you ever heard about HPV?”, the score for the PO subscale increased by 0.21. The variables explained 32% of the PB subscale score (F = 58.487; *p* < 0.000), and the best positive predictor was “Have you ever heard about HPV?”. For every one-unit increase in the case of hearing about HPV before this study, the score for the PB subscale increased by 0.46. The variables explained 13% of the Pe S subscale score (F = 18.852; *p* < 0.000). Among all of these variables, and using the β coefficient, “Have you ever heard about HPV?” was the best positive predictor. For every one-unit increase in “Have you ever heard about HPV?”, the score for the Pe S subscale increased by 0.34 (Table 3).

### 3.5. Results of Multiple Linear Regression Analysis for the Factors Affecting Students’ HPV Knowledge Levels

In the study, multiple regression analysis was used to determine the predictive capacity of independent variables (age, gender, department, employment status, hearing about HPV before this study, and HPV vaccination status) according to the four dimensions of the HPV-KS. The results of the analyses are shown in Table 4. The variables explained 51% of the overall HPV knowledge subscale score (F = 125.615; *p* < 0.000), and the best positive predictor was “Have you ever heard about HPV?”. For every one-unit increase in “Have you ever heard about HPV?”, the score for the overall HPV knowledge sub-dimension increased by 0.54. The variables explained 23% of the HPV screening test knowledge subscale score (F = 36.704; *p* < 0.000). Among all of these variables, and using the β coefficient, “Have you ever heard about HPV?” was the best positive predictor. For every one-unit increase in “Have you ever heard about HPV?”, the score for the HPV screening test knowledge subscale increased by 0.24. The variables explained 33% of the overall HPV vaccine knowledge subscale score (F = 60.124; *p* < 0.000), and the best positive predictor was “Have you ever heard about HPV?”. For every one-unit increase in “Have you ever heard about HPV?”, the general HPV vaccine knowledge subscale score increased by 0.36. The variables explained 15% of the current HPV vaccination program knowledge subscale score (F = 21,686; *p* < 0.000). Among all of these variables, and using the β coefficient, “Have you ever heard about HPV?” was the best positive predictor. For every one-unit increase in “Have you ever heard about HPV?”, the score for the current HPV vaccination program knowledge increased by 0.26 (Table 4).

## 4. Discussion

This study aimed to determine the health beliefs and levels of knowledge about HPV infection and the vaccine in students and to reveal the relationship between sociodemographic variables, health beliefs, and knowledge levels.

### 4.1. Comparison of Health Beliefs about HPV Infection/Vaccination and HPV Knowledge Levels according to Sociodemographic Characteristics

In the study, sociodemographic variables were compared with the scale mean scores. The HBMS-HPVV subscale mean scores of the female students (PS, PB, Pe S) were found to be higher than the mean scores of the male students, and a statistically significant difference was determined only with the PB subscale. Moreover, the HPV-KS subscale mean scores of the female students were found to be higher compared to the scores of the male students, and a statistically significant difference was determined. In studies conducted in Turkey and in the world on HPV infection and the vaccine, it was determined that women had more knowledge about HPV infection and the vaccine than men [19,20,21,22]. As public health vaccination campaigns mostly highlight the vaccine as a preventive measure against cervical cancer, many men see HPV as a women’s problem. It is thought that this is also the reason women have more knowledge about the infection. However, other than in cases of cervical cancer, the prevalence of HPV infection in men is a little higher than in women, and related cancers (anal and oropharyngeal) are more prevalent and increasing among men [23]. Accordingly, education programs on HPV infection and the vaccine should focus particularly on male students. 

It was determined in the study that PTR students’ HPV-KS and HBMS-HPVV mean scores were significantly lower. The results of another study conducted in Turkey support the results of the present study [15]. The nursing and midwifery students participating in the present study receive education on cervical cancer and the HPV vaccine in the courses they take in their second, third, and fourth years of study. Therefore, it is only natural for PTR students to have scored low on both scales. It is thought that the lack of education about HPV infection and the vaccine in their courses may have affected the responses of these students. 

The percentage of students who had heard of HPV infection prior to taking part in the current study was 71.6%. Nevertheless, it is surprising that some students at university level had never previously heard of HPV infection. In various studies, the rate of the participants who had heard of HPV infection varied between 16.8% and 100% [16,17,19,24,25]. In the present study, the HBMS-HPVV subscale mean scores and HPV-KS subscale mean scores of the students who had heard of HPV were determined to be higher than the mean scores of those who had not, and a statistically significant difference was determined (*p* < 0.001). It is believed that this situation resulted from the students’ previous knowledge about the negative consequences of HPV infection, their having awareness about it, and their sensitivity and the fact that they took the disease seriously.

The knowledge about the current HPV vaccination program subscale mean score of the students who were not employed was found to be higher than that of students who did work. This is thought to be due to the unemployed students being able to research more information about HPV infection due to having more free time. 

In the study, the HBMS-HPVV subscale (PS, PB, and Pe S) mean scores of the students who were vaccinated against HPV were found to be higher than those of the students who were not vaccinated, and a statistically significant difference was found only with the subscales of PS and Pe S. The results showed that the students who were not vaccinated scored higher on the PO subscale, but there was no statistically significant difference. In addition, the HPV-KS subscale mean scores of the students who were vaccinated were found to be higher, and a statistically significant difference was determined only with the subscale of general HPV knowledge. This is thought to have resulted from the fact that the students who were vaccinated had awareness of the negative consequences of HPV, that they took the disease more seriously, and that they displayed sensitivity in this regard. The high score on the PO subscale shows that the obstacles to vaccination are thought to be high in number. In parallel with the scale’s evaluation criterion, the PO scores of the students who were not vaccinated were found to be high in the present study. The students who participated in the present study came from different regions of the country to study at the university and had different cultural backgrounds. It is thought that these students from different areas and cultural traditions may have faced more obstacles. In support of the present study, it has been emphasized that religion and culture have an important effect on perceptions and knowledge of, and attitude towards, the HPV vaccine [26]. 

In addition, the HPV-KS subscale mean scores of the students who were vaccinated were found to be higher, and a statistically significant difference was determined only for the subscale of general HPV knowledge. However, the HPV vaccination rate among the students in the present study was found to be 6.9%, which is very low. In a study conducted in Turkey in 2016 on young women between the ages of 18 and 22, it was determined that only 1.3% of the participants were vaccinated against HPV [17]. A similar study in Turkey found the HPV vaccination rate among students to be 1.5% [19]. The higher rate found in the present study is promising, but it is not at a desired level. Vaccination rates are higher in countries which have national HPV vaccination programs. Within the scope of a vaccination program in South Korea, 80.6% of girls at the age of 11 years and 20.6% of girls at the age of 12 years were vaccinated with the HPV vaccine [20]. In a study in the USA, 15.8% of male and 47.3% of female students were vaccinated [27]. The WHO estimates that 15% of females globally have had their first dose of HPV [7]. The human papillomavirus (HPV) vaccine aims to minimize or even completely eradicate cervical cancer [28]. Randomized controlled trials have demonstrated that HPV vaccinations are effective. The vaccine’s effectiveness against cervical intraepithelial neoplasia (CIN2+), one of the kinds of HPV, is up to 99% in women who have never had HPV [29]. According to a study, women who received the vaccine prior to sexual intercourse had less severe cytological and colposcopy findings than those who had not received the vaccine [30]. HPV vaccination significantly lowers the chance of CIN2+ in females if administered before sexual life begins. In women who are HPV-naive, HPV immunizations are more effective [31]. The present study revealed a lack of knowledge about the HPV vaccine even among health sciences students. Although the development of the HPV vaccine has been a significant step forward in science, very few of the participants of the study were vaccinated against HPV. Students should thus be given more information about HPV infection and vaccination. This low rate of HPV vaccination determined in the study could be the result of inadequate knowledge of HPV infection and the vaccine, the high cost of the vaccine, and absence of free HPV vaccine administration in the national immunization program. Therefore, national policies that will completely or partially subsidize a better organized and sustainable awareness-raising program and HPV vaccination program should be developed. 

### 4.2. Health Beliefs of Students Regarding HPV Infection/Vaccination and HPV Knowledge Levels

The HPV infection/vaccine knowledge level mean scores and health belief level mean scores were evaluated. The results showed that the students had low levels of knowledge about HPV infection and the vaccine. In other studies conducted in Turkey and other countries, HPV knowledge levels were also found to be low [17,24,32,33]. In this sense, the results of this study are consistent with the literature. Regarding the mean scores obtained from the HBMS-HPVV, the students’ mean scores for the subscales of PS, PB, and Pe S were found to be a little over the average, while their mean scores for the subscale of PO were found to be lower than the average. Similar to the present study, in other studies conducted in the literature the participants’ PO scores were found to be low, while their scores for the subscales of PS, PB, and PeS were high [15,17,34]. In order to increase knowledge about the HPV vaccine, it is important to educate students about its existence and to reach them by organizing vaccine awareness programs at university. 

### 4.3. Multiple Linear Regression Analysis of Factors Influencing Students’ Health Beliefs about HPV Infection/Vaccination and HPV Knowledge Levels

According to the multilinear regression analysis performed, the main determinant of the scores for PS, PO, Pe S, and PB subscales of the HBMS-HPVV was the state of having heard of HPV before. Similarly, it was determined that the main determinant of the scores for the HPV-KS, general HPV screening test knowledge, general HPV vaccine knowledge, and current HPV vaccination program knowledge was the state of having heard of HPV before. In one study, it was revealed that HPV vaccine knowledge and perceived HPV risk and degree were significant predictors of the intention to get vaccinated against HPV [35]. In line with these results, it is thought that a high level of knowledge about HPV among students positively affects their health beliefs regarding HPV. 

### 4.4. Limitations of the Study

This study sampled health science students from a single university. Therefore, our results cannot be generalized to all health science students in Turkey. Further studies targeting other university students in Turkey are recommended. Despite this limitation, the study was able to make predictions about students’ knowledge and beliefs about HPV infection and the vaccine.

## 5. Conclusions

The participants of the study were found to have a low level of knowledge about HPV, and their awareness of the HPV vaccine was low. The results of the study show that regardless of their background, more aggressive educational activities for university students about HPV infection are needed, as is greater promotion of the vaccine. It is thus recommended to diversify education curricula in order to provide healthcare students with knowledge about HPV infection and the vaccine, as well as initiating periodic screenings so that HPV-related morbidity and mortality can be decreased. In addition, sustainable awareness programs and national policies to fully subsidize the HPV vaccination program should be implemented to increase the rate of vaccination.

## Figures and Tables

**Table 1:** Characteristics of the participants and differences in means based on human papillomavirus infection and vaccine health belief model and human papillomavirus knowledge.

Characteristics	N	%	HBMS-HPVV				HPV-KS			
			Perceived Severity Mean ± SD	Perceived Obstacle Mean ± SD	Perceived Benefit Mean ± SD	Perceived Sensitivity Mean ± SD	General HPV Knowledge Mean ± SD	HPV Screening Test Knowledge Mean ± SD	General HPV Vaccine Knowledge Mean ± SD	Knowledge of Current HPV Vaccination Program Mean ± SD
**Mean age (yrs)**							**20.92 ± 1.60**			
Gender										
Female	677	82.2	2.52 ± 0.85	1.87 ± 0.59	2.72 ± 0.90	2.35 ± 0.81	7.50 ± 4.05	1.66 ± 1.55	2.27 ± 1.69	1.36 ± 1.39
Male	147	17.8	2.38 ± 0.83	1.93 ± 0.71	2.42 ± 0.84	2.33 ± 0.83	5.72 ± 3.89	1.06 ± 1.19	1.34 ± 1.39	1.07 ± 1.36
			t = 1.924; *p* = 0.055	t = −1.119; *p* = 0.263	t = 3.633; *p* = 0.000 **	t = 0.228; *p* = 0.820	t = 4.865; *p* =0.000 **	t = 4.351; *p* = 0.000 *	t = 6.225; *p* = 0.000 **	t = 2.134; *p* = 0.021 *
Department										
Nursing	450	54.6	2.43 ± 0.85	1.92 ± 0.68	2.57 ± 0.87	2.34 ± 0.84	6.62 ± 3.80	1.29 ± 1.32	1.77 ± 1.50	1.16 ± 1.31
Midwifery	267	32.4	2.77 ± 0.73	1.88 ± 0.47	3.05 ± 0.76	2.44 ± 0.70	9.50 ± 3.21	2.26 ± 1.71	3.08 ± 1.60	1.79 ± 1.48
Physical Therapy and Rehabilitation	107	13.0	2.11 ± 0.92	1.72 ± 0.62	2.11 ± 0.90	2.13 ± 0.94	3.79 ± 3.99	0.87 ± 0.96	1.03 ± 1.35	0.76 ± 1.12
			F = 27.260; *p* = 0.000 **, c < a,b *	F = 4.290; *p* = 0.014 *, c < a *	F = 54.023; *p* = 0.000 **, c < a,b *	F = 5.600; *p* = 0.004 *, c < a,b *	F = 105.458; *p* = 0.000 **, c < a,b *	F = 52.343; *p* = 0.000 **, c < a,b *	F = 93.464; *p* = 0.000 **, c < a,b *	F = 2.492; *p* = 0.000 **, c < a,b *
Working status										
Yes	77	9.3	2.44 ± 0.89	1.97 ± 0.75	2.50 ± 1.02	2.37 ± 0.93	7.22 ± 4.08	1.66 ± 1.72	2.22 ± 1.72	0.94 ± 1.27
No	747	90.7	2.50 ± 0.84	1.87 ± 0.60	2.68 ± 0.88	2.34 ± 0.80	7.18 ± 4.08	1.54 ± 148	2.09 ± 1.67	1.35 ± 1.40
			t = −0.668; *p* = 0.504	t = 1.257; *p* = 0.209	t = −1.732; *p* = 0.084	t = 0.217; *p* = 0.828	t = 0.076; *p* = 0.939	t = 0.656; *p* = 0.512	t = 0.639; *p* = 0.523	t = −2.435; *p* = 0.015 *
Have you ever heard about HPV?										
Yes	590	71.6	2.76 ± 0.69	1.97 ± 0.56	2.97 ± 0.70	2.53 ± 0.71	8.89 ± 3.04	1.91 ± 1.54	2.62 ± 1.60	1.60 ± 1.42
No	234	28.4	1.83 ± 0.84	1.67 ± 0.69	1.89 ± 0.85	1.87 ± 0.87	2.87 ± 3.07	0.63 ± 0.92	0.80 ± 1.02	0.80 ± 1.02
			t = 16.245; *p* = 0.000 *	t = 6.421; *p* = 0.000 **	t = 18.568; *p* = 0.000 **	t = 11.264; *p* = 0.000 **	t =25.574; *p* = 0.000 *	t = 11.870; *p* = 0.000 **	t = 16.037; *p* = 0.000 **	t = 9.883; *p* = 0.000 **
Vaccinated against HPV										
Yes	57	6.9	2.75 ± 0.73	1.82 ± 0.51	2.89 ± 0.65	2.63 ± 0.74	8.29 ± 2.58	1.85 ± 1.43	2.24 ± 1.58	2.05 ± 1.48
No	767	93.1	2.48 ± 0.85	1.89 ± 0.62	2.65 ± 0.91	2.32 ± 0.82	7.10 ± 4.16	1.53 ± 1.51	2.09 ± 1.68	1.26 ± 1.37
			t = 2.352; *p* = 0.019 *	t = −0.786; *p* = 0.432	t = 1.955; *p* = 0.051	t = 2.685; *p* = 0.007 *	t = 29.015; *p* = 0.000 *	t = 1.034; *p* = 0.310	t = 1.433; *p* = 0.232	t = 0.169; *p* = 0.681
Marital status										
Single	824	100.0								

Abbreviations: HBMS-HPVV, human papillomavirus infection and vaccine health belief model scale; HPV-KS, human papillomavirus knowledge scale; *p*, level of statistical significance; a: Nursing; b: Midwifery; c: Physical Therapy and Rehabilitation; F = ANOVA; t = Student’s *t* test; * *p* < 0.05, ** *p* < 0.001.

**Table 2:** Descriptive statistics for the total number of scale.

Variable	Subscales	Mean ± SD	Min–Max	Items	Skewness	Kurtosis
HPV-KS	General HPV knowledge	7.88 ± 4.08	0–15	16	−0.306	−1.209
	HPV screening test knowledge	1.55 ± 1.51	0–6	6	0.846	−0.141
	General HPV vaccine knowledge	2.10 ± 1.67	0–5	5	0.204	−1.273
	Knowledge of current HPV vaccination program	1.31 ± 1.39	0–6	6	0.816	−0.0400
	Total score	12.16 ± 7.21	0–27	33	−0.045	−1.134
HBMS-HPVV	Perceived severity	2.50 ± 0.85	1–4	4	−0.264	−0.698
	Perceived obstacle	1.88 ± 0.62	1–4	5	0.513	0.329
	Perceived benefit	2.67 ± 0.89	1–4	3	−0.381	−0.677
	Perceived sensitivity	2.35 ± 0.82	1–4	2	0.072	−0.553
	Total score	32.16 ± 8.91	14–56	14	−0.551	0.108

Abbreviations: HBMS-HPVV, human papillomavirus infection and vaccine health belief model scale; HPV-KS, human papillomavirus knowledge scale; SD, standard deviation.

**Table 3:** Multiple linear regression model for predicting human papillomavirus infection/vaccine health belief and sociodemographic data.

		Unstandardized Coefficients		Standardized Coefficients			95% CI		
Variable	Model	B	SD	β	t	*p*	Lower	Upper	R^2^
Perceived severity	Constant	0.322	0.37		0.860	0.390	−0.413	1.058	0.26
	Age	0.069	0.01	0.12	3.980	0.000 *	0.035	0.103	
	Cinsiyet (reference: boy)	−0.011	0.07	−0.00	−0.155	0.877	−0.152	0.129	
	Department (reference: Physiotherapy and Rehabilitation)								
	Nurse	0.036	0.82	0.02	0.444	0.657	−0.124	0.197	
	Midwifery	0.205	0.09	0.11	2.243	0.025 *	0.026	0.384	
	Working status (reference: no)	0.094	0.08	0.03	1.064	0.287	−0.080	0.268	
	Have you ever heard about HPV? (reference: no)	0.799	0.06	0.42	12.723	0.000 *	0.676	0.922	
	HPV vaccination status (reference: no)	0.126	0.10	0.10	1.222	0.222	−0.076	0.329	
Perceived obstacle	Constant	1.057	0.30		3.436	0.001 *	0.453	1.660	0.06
	Age	0.034	0.01	0.08	2.380	0.018 *	0.006	0.062	
	Cinsiyet (reference: boy)	−0.072	0.05	−0.04	−1.230	0.219	−0.188	0.043	
	Department (reference: Physiotherapy and Rehabilitation)								
	Nurse	0.083	0.06	0.06	1.232	0.218	−0.049	0.214	
	Midwifery	−0.010	0.07	−0.00	−0.138	0.890	−0.158	0.137	
	Working status (reference: no)	−0.064	0.07	−0.03	−0.877	0.381	0.207	0.079	
	Have you ever heard about HPV? (reference: no)	0.296	0.05	0.21	5.739	0.000 *	0.195	0.397	
	HPV vaccination status (reference: no)	−0.167	0.08	−0.06	−1.975	0.049 *	−0.334	−0.001	
Perceived benefit	Constant	0.762	0.37		2.023	0.043 *	0.023	1.502	0.32
	Age	0.038	0.01	0.06	2.171	0.030 *	0.004	0.072	
	Cinsiyet (reference: boy)	0.058	0.07	0.02	0.806	0.421	−0.083	0.199	
	Department (reference: Physiotherapy and Rehabilitation)								
	Nurse	0.188	0.08	0.10	2.290	0.022 *	0.027	0.349	
	Midwifery	0.460	0.09	0.24	5.003	0.000 *	0.279	0.640	
	Working status (reference: no)	0.170	0.08	0.05	1.912	0.056	−0.005	0.345	
	Have you ever heard about HPV? (reference: no)	0.920	0.06	0.46	14.570	0.000 *	0.796	1.044	
	HPV vaccination status (reference: no)	0.106	0.10	0.03	1.019	0.309	−0.098	0.310	
Perceived sensitivity	Constant	1.410	0.39		3.598	0.000 *	0.641	2.179	0.13
	Age	0.025	0.01	0.04	1.363	0.173	−0.011	0.060	
	Cinsiyet (reference: boy)	−0.058	0.07	−0.02	−0.780	0.432	−0.205	0.089	
	Department (reference: Physiotherapy and Rehabilitation)								
	Nurse	0.017	0.08	0.01	0.203	0.839	−0.150	0.185	
	Midwifery	0.012	0.09	0.07	0.121	0.904	−0.176	0.199	
	Working status (reference: no)	−0.004	0.09	−0.02	−0.048	0.962	−0.186	0.178	
	Have you ever heard about HPV? (reference: no)	0.635	0.06	0.34	9.661	0.000 *	0.506	0.763	
	HPV vaccination status (reference: no)	0.144	0.108	0.04	1.337	0.182	−0.068	0.356	

Note: * *p* is significant at the *p* < 0.05 level. Abbreviations: B, unstandardized regression coefficients; adjusted R^2^, variance explained; t, Student’s *t* test; β, standardized regression coefficients.

**Table 4:** Multiple linear regression model for predicting human papillomavirus knowledge and sociodemographic data.

		Unstandardized Coefficients		Standardized Coefficients			95% CI		
Variable	Model	B	SD	β	t	*p*	Lower	Upper	R^2^
General HPV knowledge	Constant	−0.3.917	1.45		−2.688	0.007 *	−6.777	−1.057	0.51
	Age	0.265	0.06	0.10	3.953	0.000 *	0.134	0.397	
	Cinsiyet (reference: boy)	0.455	0.27	0.04	1.635	0.102	−0.091	1.001	
	Department (reference: Physiotherapy and Rehabilitation)								
	Nurse	1.329	0.31	0.16	4.182	0.000 *	0.705	1.952	
	Midwifery	3.058	0.35	0.35	8.606	0.000 *	2.360	3.755	
	Working status (reference: no)	−0.131	0.34	−0.00	−0.379	0.705	−0.807	0.546	
	Have you ever heard about HPV? (reference: no)	4.944	0.24	0.54	20.247	0.000 *	4.464	5.423	
	HPV vaccination status (reference: no)	0.570	0.40	0.03	0.156	0.156	−0.218	1.358	
HPV screening test knowledge	Constant	−3.373	0.67		−4.975	0.000 *	−4.703	−2.042	0.23
	Age	0.186	0.03	0.19	5.968	0.000 *	0.125	0.248	
	Cinsiyet (reference: boy)	0.275	0.12	0.07	2.123	0.034 *	0.021	0.529	
	Department (reference: Physiotherapy and Rehabilitation)								
	Nurse	0.028	0.14	0.00	0.190	0.850	−0.262	0.318	
	Midwifery	0.735	0.16	0.22	4.444	0.000 *	0.410	1.059	
	Working status (reference: no)	−0.081	0.16	−0.01	0.505	0.613	−0.396	0.234	
	Have you ever heard about HPV? (reference: no)	0.835	0.11	0.24	7.348	0.000 *	0.612	1.058	
	HPV vaccination status (reference: no)	0.341	0.18	0.05	1.825	0.068	−0.026	0.708	
General HPV vaccine knowledge	Constant	−1.567	0.70		−2.234	0.026 *	−2.943	−0.190	0.33
	Age	0.093	0.03	0.08	2.889	0.004 *	0.030	0.157	
	Cinsiyet (reference: boy)	0.400	0.13	0.09	2.990	0.003 *	0.138	0.663	
	Department (reference: Physiotherapy and Rehabilitation)								
	Nurse	0.329	0.15	0.09	2.153	0.032 *	0.029	0.629	
	Midwifery	1.252	0.17	0.34	7.320	0.000 *	0.916	1.587	
	Working status (reference: no)	−0.194	0.16	−0.03	−1.167	0.243	−0.519	0.132	
	Have you ever heard about HPV? (reference: no)	1.354	0.11	0.36	11.524	0.000 *	1.124	1.585	
	HPV vaccination status (reference: no)	0.131	0.19	0.02	0.679	0.498	−0.248	0.511	
Knowledge of current HPV vaccination program	Constant	0.819	0.65		1.243	0.214	−0.474	2.112	0.15
	Age	−0.037	0.03	−0.04	−1.216	0.224	−0.096	0.023	
	Cinsiyet (reference: boy)	−0.009	0.12	−0.00	−0.073	0.942	−0.256	0.283	
	Department (reference: Physiotherapy and Rehabilitation)								
	Nurse	0.212	0.14	0.07	1.475	0.141	−0.070	0.494	
	Midwifery	0.730	0.16	0.24	4.546	0.000 *	0.415	1.045	
	Working status (reference: no)	0.321	0.15	0.06	2.060	0.040 *	0.015	0.627	
	Have you ever heard about HPV? (reference: no)	0.813	0.11	0.26	7.368	0.000 *	0.597	1.030	
	HPV vaccination status (reference: no)	0.731	0.18	0.13	4.029	0.000 *	0.375	1.088	

Note: * *p* is significant at the *p* < 0.05 level. Abbreviations: B, unstandardized regression coefficients; adjusted R^2^, variance explained; t, Student’s t test; β, standardized regression coefficients.

## Data Availability

The data presented in this study are available on request from the corresponding author.

## References

[1] World Health Organization (2017). Human Papillomavirus Vaccines: WHO Position Paper, May 2017. Wkly. Epidemiol. Rec..

[2] Meites E., Gee J., Unger E., Markowitz L., Hall E., Wodi A.P., Hamborsky J., Morelli V., Schillie S. (2021). Human Popillomavirus. Epidemiology and Prevention of Vaccine-Preventable Diseases.

[3] Bruni L., Albero G., Serrano B., Mena M., Collado J.J., Gómez D., Muñoz J., Bosch F.X., de Sanjosé S. (2023). Human Papillomavirus and Related Diseases in Turkey.

[4] World Health Organization (2020). Global Strategy to Accelerate the Elimination of Cervical Cancer as a Public Health Problem.

[5] Bora Başara B., Aygün A., Soytutan Çağlar İ., Kulali B. (2021). Health Statistics Yearbook 2020 Newsletter.

[6] Senkomago V., Duran D., Loharikar A., Hyde T.B., Markowitz L.E., Unger E.R., Saraiya M. (2017). CDC Activities for Improving Implementation of Human Papillomavirus Vaccination, Cervical Cancer Screening, and Surveillance Worldwide. Emerg. Infect. Dis..

[7] World Health Organization (2021). Immunization Coverage. https://www.who.int/news-room/fact-sheets/detail/immunization-coverage.

[8] Vaidakis D., Moustaki I., Zervas I., Barbouni A., Merakou K., Chrysi M.S., Creatsa G., Panoskaltsis T. (2017). Knowledge of Greek adolescents on Human Papilloma Virus (HPV) and vaccination: A national epidemiologic study. Medicine.

[9] Akalın A. (2021). Human Papillomavirus (HPV) Infection and current approaches to HPV vaccine. Androl Bul..

[10] Markowitz L.E., Dunne E.F., Saraiya M., Chesson H.W., Curtis C.R., Gee J., Bocchini J.A., Unger E.R. (2014). Human Papillomavirus vaccination: Recommendations of the Advisory Committee on Immunization Practices (ACIP). Morb. Mortal. Wkly. Rep..

[11] Petrosky E., Bocchini J.A., Hariri S., Chesson H., Curtis C.R., Saraiya M., Unger E.R., Markowitz L.E., Centers for Disease Control and Prevention (CDC) (2015). Use of 9-valent human papillomavirus (HPV) vaccine: Updated HPV vaccination recommendations of the advisory committee on immunization practices. Morb. Mortal. Wkly. Rep..

[12] Fontenot H.B., Collins Fantasia H., Charyk A., Sutherland M.A. (2014). Human papillomavirus (hpv) risk factors, vaccination patterns, and vaccine perceptions among a sample of male college students. J. Am. Coll. Health.

[13] Cheung T., Lau J.T.F., Wang J.Z., Mo P.K.H., Ho Y.S. (2018). Acceptability of HPV vaccines and associations with perceptions related to HPV and HPV vaccines among male baccalaureate students in Hong Kong. PLoS ONE.

[14] Duval B., Gilca V., Boulianne N.V., Pielak K., Halperin B., Simpson M.A., Sauvageau C., Ouakki M., Dube E., Lavoie F. (2009). Cervical cancer prevention by vaccination: Nurses’ knowledge, attitudes and intentions. J. Adv. Nurs..

[15] Altıntaş R.Y., Erciyas Ş.K., Ertem G. (2022). Determination of health belief levels of faculty of health sciences students regarding cervical cancer and human papilloma virus ınfection vaccination. E-J. Dokuz Eylul Univ. Nurs. Fac..

[16] Khatiwada M., Kartasasmita C., Mediani H.S., Delprat C., Van Hal G., Dochez C. (2021). Knowledge, attitude and acceptability of the human papilloma virus vaccine and vaccination among university students in Indonesia. Front. Public Health.

[17] Güvenç G., Seven M., Akyüz A. (2016). Health belief model scale for human papilloma virus and its vaccination: Adaptation and psychometric testing. J. Pediatr. Adolesc. Gynecol..

[18] Demir F. (2019). Validity and Reliability of the Turkish Version of Human Papilloma Virus Knowledge Scale. Master’s Thesis.

[19] Çınar İ.O., Ozkan S., Aslan G.K., Alatas E. (2019). Knowledge and behavior of university students toward human papillomavirus and vaccination. Asia Pac. J. Oncol. Nurs..

[20] Kim S.Y., Seo J.W., Ryu E. (2021). Korean college students’ attitudes and health behaviour regarding human papillomavirus vaccination. Collegian.

[21] Du E.Y., Adjei Boakye E., Taylor D.B., Kuziez D., Rohde R.L., Pannu J.S., Simpson M.C., Patterson R.H., Varvares M.A., Osazuwa-Peters N. (2022). Medical students’ knowledge of HPV, HPV vaccine, and HPV-associated head and neck cancer. Hum. Vaccines Immunother..

[22] Galvão M.P.S.P., Araújo T.M.E., Rocha S.S. (2022). Knowledge, attitudes, and practices of adolescents regarding human papillomavirus. Rev. Saude Publica.

[23] Serrano B., Brotons M., Bosch F.X., Bruni L. (2018). Epidemiology and burden of HPV-related disease. Best Pract. Res. Clin. Obstet. Gynaecol..

[24] Suhaila K., Mukherjee A., Maharjan B., Dhakal A., Lama M., Junkins A., Khakurel U., Jha A.N., Jolly P.E., Lhaki P. (2021). Human papillomavirus, related diseases, and vaccination: Knowledge and awareness among health care students and professionals in Nepal. J. Cancer Educ..

[25] Ramesh P.S., Krishnamurthy S., Shrestha S., Nataraj S.M., Devegowda D. (2021). Knowledge, awareness and prevalence of human papillomavirus among local university students and healthcare workers in South India: A cross-sectional study. Clin. Epidemiol. Glob. Health.

[26] Ersin F., Kıssal A., Polat P., Koca B.D., Erdoğan M. (2016). Perception of female medical personnel toward cervical cancer and the affecting factors. J. Res. Dev. Nurs..

[27] Barnard M., George P., Perryman M.L., Wolff L.A. (2017). Human papillomavirus (HPV) vaccine knowledge, attitudes, and uptake in college students: Implications from the precaution adoption process model. PLoS ONE.

[28] Hall M.T., Simms K.T., Lew J.B., Smith M.A., Brotherton J.M., Saville M., Frazer I.H., Canfell K. (2019). The projected timeframe until cervical cancer elimination in australia: A modelling study. Lancet Public Health.

[29] Arbyn M., Xu L., Simoens C., Martin-Hirsch P.P. (2018). Prophylactic vaccination against human papillomaviruses to prevent cervical cancer and its precursors. Cochrane Database Syst. Rev..

[30] Paraskevaidis E., Athanasiou A., Paraskevaidi M., Bilirakis E., Galazios G., Kontomanolis E., Dinas K., Loufopoulos A., Nasioutziki M., Kalogiannidis I. (2020). Cervical Pathology Following HPV Vaccination in Greece: A 10-year HeCPA Observational Cohort Study. In Vivo.

[31] Szarewski A., Poppe W.A., Skinner S.R., Wheeler C.M., Paavonen J., Naud P., Salmeron J., Chow S.N., Apter D., Kitchener H. (2012). Efficacy of the human papillomavirus (HPV)-16/18 as04-adjuvanted vaccine in women aged 15–25 years with and without serological evidence of previous exposure to HPV-16/18. Int. J. Cancer.

[32] D’Errico M.P., Tung W.C., Lu M., D’Errico R. (2020). Knowledge, attitudes, and practices related to human papillomavirus vaccination among college students in a state university: Implications for nurse practitioners. J. Am. Assoc. Nurse Pract..

[33] Park A.S. (2015). The influence of cervical cancer, HPV knowledge and health beliefs on HPV vaccination among undergraduate students. J. Korea Acad.-Ind. Coop. Soc..

[34] Kim H.W. (2012). Knowledge about human papillomavirus (HPV) and health beliefs and intention to recommend HPV vaccination for girls and boys among Korean health teachers. Vaccine.

[35] Xu Y., Bi W., Liu T., Jiang Y., Wang Q., Fan R. (2021). Factors associated with intention of human papillomavirus vaccination among Chinese college students: İmplications for health promotion. Hum. Vaccines Immunother..

